# The bidirectional associations between sarcopenia-related traits and cognitive performance

**DOI:** 10.1038/s41598-024-58416-w

**Published:** 2024-03-31

**Authors:** Chun-feng Lu, Wang-shu Liu, Xiao-min Cang, Xin Sun, Xue-qin Wang, Chun-hua Wang, Feng Xu

**Affiliations:** 1 Department of Endocrinology, Nantong City No 1 People’s Hospital and Second Affiliated Hospital of Nantong University, No. 666 Shengli Road, Nantong, 226001 China; 2 Department of Anesthesiology, Nantong City No 1 People’s Hospital and Second Affiliated Hospital of Nantong University, No. 666 Shengli Road, Nantong, 226001 China

**Keywords:** Neuroscience, Neurology

## Abstract

While many studies have sought to explore the degree to which sarcopenia-related traits are associated with cognitive performance, these studies have yielded contradictory results without any clear indication of the causality of such relationships. In efforts to better understand associations between sarcopenia-related traits and cognitive ability, a series of multivariate linear regression assessments were carried out upon datasets derived through the National Health and Nutrition Examination Survey (NHANES). Of these, cognitive performance was assessed by the Digit Symbol Substitution Test (DDST), the Consortium to Establish a Registry for Alzheimer’s Disease Immediate Recall Test (CERAD-IR), Delayed Recall Test (CERAD-DR) and Animal Fluency Test (AFT). Causal relationships between the two were further inferred via a two-sample Mendelian randomization (MR) analysis approach. Sarcopenia-related traits considered in these assessments included walking speed, appendicular skeletal muscle mass (ASM), and hand grip strength (HGS). Walking speed, ASM, and HGS were all significantly independently related to cognitive scores following adjustment for covariates. MR assessments also identified that each 1-SD higher walking speed and appendicular lean mass were causally and respectively associated with a 0.34 [standard error (SE) = 0.09; *p* < 0.001)] standardized score higher and a 0.07 (SE = 0.01; *p* < 0.001) standardized score higher cognitive score, whereas a higher hand grip strength was positively associated with a better cognitive performance. Reverse MR assessments also yielded similar findings. These data suggest that lower walking speed, muscle strength, and muscle mass were all closely related to lower cognitive performance irrespective of gender, and that there may be a mutually reinforcing relationship among these variables.

## Introduction

As life expectancy increases, the proportion of older adults in the general population continues to rise steadily. There were an estimated 1 billion individuals ≥ 60 years of age in 2020, and this number is forecast to rise towards 2.1 billion by 2050^1^. Health and quality of life of these older adults remains a pressing global public health issue. Hallmarks of aging include both cognitive decline and sarcopenia, the latter of which affects up to 33.0% of older adults^2^. Sarcopenia is closely tied to a range of adverse outcomes that include falls, fractures, disability, and death^2^. Cognition function including memory, planning, reasoning, and/or language competences is essential for maintaining independence in daily living^3^. Mild cognitive impairment (MCI), recognized as a clinical syndrome harbinger of dementia, affects about 6% of people aged 60–64 and 25% of people aged 80–84^4^. About 5% to 10% of those with MCI will progress to dementia annually^5^. Sarcopenia and cognitive performance decline share multiple pathophysiological mechanisms, such as aging, reduced activity, neuromuscular injury, insulin resistance, hormone dysregulation, oxidative stress, chronic inflammation and so on^6,7^. Given the overlapping pathogenesis and often concurrent pathogenesis of sarcopenia and cognitive decline, studies exploring the associations between these two conditions represent a major hotspot of ongoing research interest.

One recent meta-analysis of data derived from 15 cross-sectional investigations explored associations between sarcopenia and MCI within older adults and ultimately revealed that the presence of sarcopenia was associated with a 2.25-fold increased risk of MCI^8^. In contrast, other cross-sectional assessments conducted in France^9^ and Korea^10^ have failed to identify any significant associations between sarcopenia and cognitive decline. This may be attributable to differences in the ages of the study populations, inconsistent approaches to assessing sarcopenia or cognition, and other factors. Components of sarcopenia include decreased muscle strength, poor muscle function, and low muscle mass. In one prospective study, poor muscle function, while not low muscle mass was closely associated with decline of cognitive performance^11^. Outcomes of studies focused on the relationships between sarcopenia-associated traits and cognitive decline have tended to be inconsistent, and no such studies to date have convincingly documented the causal nature of associations between the two.

Recently, Mendelian randomization (MR) studies have emerged as an effective approach to detecting causal associations between particular exposures and clinical outcomes of interest as they provide a means of overcoming reverse causality bias. MR assessments rely on the use of genetic markers as exposure-related instrumental variables (IVs) to reduce the potential impact of any confounding factors^12^. Importantly, a wealth of data collected through large-scale genome-wide association studies (GWASs) remain available to enable these MR assessments.

Here, the associations between sarcopenia-related variables and cognitive performance were explored employing datasets stemming through National Health and Nutrition Examination Survey (NHANES). Moreover, such causal nature for these associations was evaluated through a two-sample bidirectional MR study.

## Materials and methods

### Study population

The Centers for Disease Control and Prevention developed the NHANES public database for compiling health- and nutrition-related data for subjects throughout the USA based on multistage, complex, and probabilistic sampling criteria^13^. This three-part cross-sectional study included an initial examination concerning the associations between walking speed and cognitive performance within individuals > 60 years of age based on data collected from 1990 to 2000 and 2001–2002. Then the associations between skeletal muscle mass index values and cognitive performance in individuals > 60 years of age were evaluated based on data collected from 2011–2012 and 2013–2014. Lastly, the relationships between hand grip strength (HGS) and cognitive performance were examined in individuals > 60 years of age based on data collected from 2011–2012 and 2013–2014.

### Sarcopenia-related trait measurements

All measurements of walking speed were taken by certified technicians at the NHANES mobile examination center. Participants were directed to walk in a straight line for 20 feet using normal speed, with timing for walking this distance was registered. Walking speed was determined by dividing 20 ft by the completion time. Prior research has demonstrated that walking speed can serve as a reliable indicator of physical performance^14^.

At present, there is no gold standard to evaluate muscle mass. Magnetic resonance imaging (MRI) is the most accurate method to evaluate muscle mass relatively. Various reasons limit the application of MRI, and DXA is the most popular method for muscle mass estimation^15^. However, there was a lack of NHANES datasets that conducted both DXA and cognitive performance test. ASM was estimated based on the algorithm provided in the previous literature and was used as an approximation for DXA^16^, and the specific algorithm was as follows: ASM = 0.193 × weight (kg) + 0.107 × height (cm) – 4.157 × gender (1-males, otherwise-0) – 0.037 × age (years) – 2.631.

HGS (kg) was assessed by a trained examiner using a Takei Digital Grip Strength Dynamometer. HGS testing was performed while patients were in a standing position, and was repeated three times per hand. Maximum HGS values for each hand were summed together and reported as the combined HGS for use in subsequent assessments^17^.

### Cognitive performance assessments

For the NHANES 1999–2002 dataset, the Digit Symbol Substitution Test (DDST) was used to evaluate cognitive performance, focusing on evaluation of sustained attention and short-term memory. In this test, each subject was given a paper form with nine numbers paired with different symbols at the paper top, and then given two minutes to copy the corresponding symbols in 133 boxes next to the numbers. The number of correct matches was used to compute the overall DSST score^18^.

For the NHANES 2011–2014 dataset, the results of four cognitive tests were used to assess cognitive performance, including the Consortium to Establish a Registry for Alzheimer’s Disease Immediate Recall Test (CERAD-IR), Delayed Recall Test (CERAD-DR), Animal Fluency Test (AFT), together with DDST^19^. In the CERAD-IR test, after being shown 10 unrelated words, participants were immediately asked to recall as many words as possible. The subjects would complete three of these word learning tests, and the sum of the results was the CERAD-IR score. The CERAD-IR and CERAD-DR tests provide a comprehensive assessment of cognitive domains. Participants received CERAD-DR test approximately 10 min after the start of the word learning trials, and the number of words recalled was the CERAD-DR score. The CERAD-total score was calculated as the CERAD-IR score plus the CERAD-DR score. In the AFT test, for testing verbal category fluency, participants were instructed to name as many animals as they could in 1 min, with one point awarded for each name. The total animals named was the AFT score.

### MR study design

**Figure 1:**
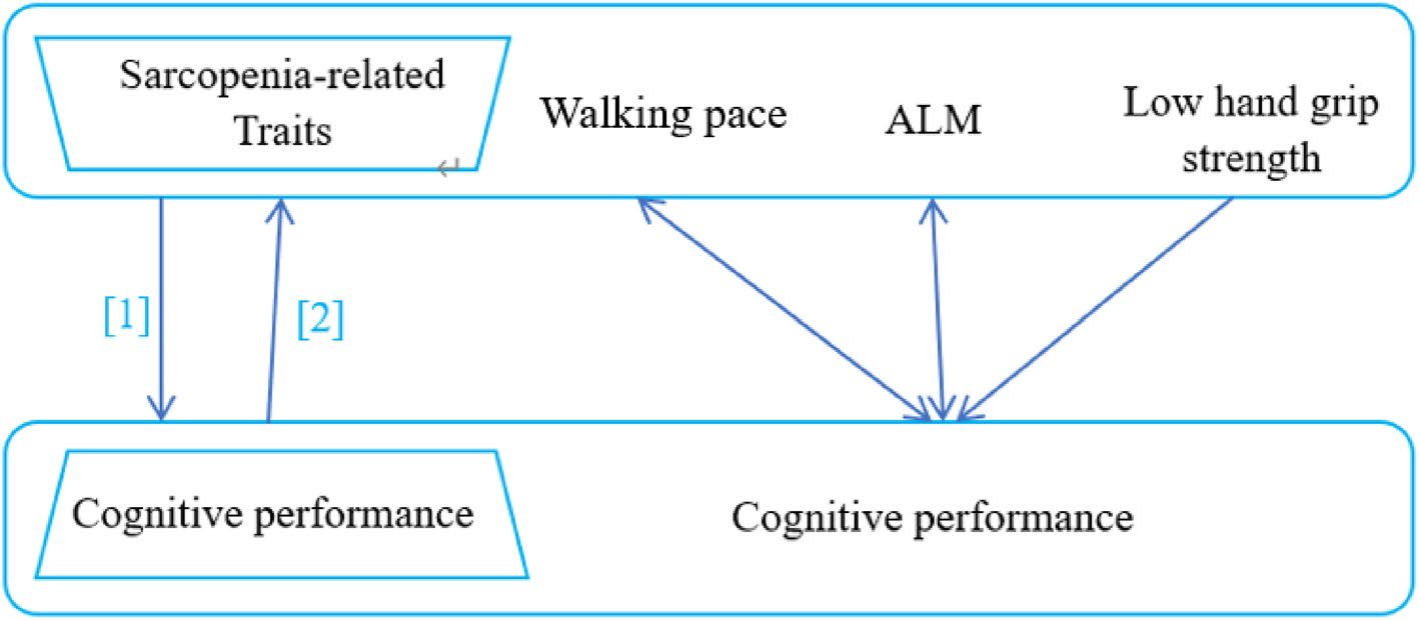
Schematic diagram of the bidirectional MR analysis. This bidirectional mendelian randomization analysis was performed in two steps: sarcopenia-related traits (walking speed, ALM, low hand grip strength) was studied as exposure while cognitive performance was studied as outcome in the first step, whereas the second step was reversed. The arrows indicate direction of causality in our results. *MR* Mendelian randomization, *ALM* appendicular lean mass.

The causality and directionality of relationships between sarcopenia-associated traits (walking speed, muscle mass, HGS) and cognitive performance were assessed through an MR study, as detailed in Fig. 1. The three key assumptions of MR are as follows: genetic variation as an instrumental variable IV must be truly correlated with exposure; genetic variation is independent of the exposure-outcome confounders; genetic variation affects outcome only through exposure.

Summary-level datasets within such an investigation stemmed through the open GWAS database maintained by the MRC Integrated Epidemiology Unit^20^, as detailed in Table 1. The UK Biobank study was the source of all data used for MR assessments, including data related to appendicular lean mass (ALM) (n = 450,243)^21^, HGS (n = 256,523)^22^, walking speed (n = 45,915), and cognitive performance (n = 257,841)^23^. For evaluating ALM, bioelectrical impedance analysis was conducted in each participant^21^. Low HGS was defined as less than 30 kg in men and less than 20 kg in women, respectively^22^. The dataset of cognitive performance included 57 population-based cohorts conducted by the Cohorts for Heart and Aging Research in Genomic Epidemiology (CHARGE), the Cognitive Genomics Consortium (COGENT) consortia, and UK Biobank^23^. In the CHARGE and COGENT cohorts, cognitive performance was evaluated by a variety of cognitive tasks focused on at least three different cognitive domains^23^. In the UK Biobank cohorts, cognitive performance was assessed by 13 multiple-choice questions^23^. These publicly accessible GWAS data were collected with appropriate ethical review board approval, and the present assessments did not require any additional approval.

**Table 1:** Phenotype source and description.

Phenotype	Consortium	Participants	Datatype	Unit	GWAS ID
Cognitive performance	UKB	257,841	Continuous	1-standarized score	ebi-a-GCST006572
Walking speed	UKB	459,915	Continuous	1-SD	ukb-b-4711
Appendicular lean mass	UKB	450,243	Continuous	1-SD	ebi-a-GCST90000025
Low hand grip strength	UKB	n = 48,956 cases and 207,927 controls	Binary	–	ebi-a-GCST90007526

Single nucleotide polymorphisms (SNPs) meeting three criteria were selected as appropriate IVs: (1) SNPs linked to exposure of interest having genome-wide significance cutoff at *p* < 5 × 10^−8^; (2) SNPs found to be independent as determined through a pairwise-linkage disequilibrium analysis^24^, with the SNP exhibiting the higher p-value or the correlation with the greatest number of other SNPs being deleted for any SNPS exhibiting an r^2^ > 0.001 (10,000 kb window); and (3) SNPs that had undergone data harmonization prior to MR assessments in order to ensure that the impact of a particular SNP on the exposure of interest was associated with the appropriate allele. The SNPs' harmonization was conducted in three steps: ensure that exposure-SNPs and outcome-SNPs strand orientation were similar; palindromic SNPs were corrected; incompatible SNPs were removed.

The primary MR analysis outcome for this study was an inverse variance-weighted (IVW) meta-analysis conducted with random-effects modeling. Additionally, weighted median and MR-Egger sensitivity assessments were also conducted, with the former offering validated predictions once > 50% dataset composition stemmed through validated IVs^25^, while latter enabling assessments of horizontal pleiotropy for IVs of interest^26^.

### Statistical analysis

NHANES data were extracted, merged, cleaned, and analyzed using R v4.2.2. A *p* value < 0.05 was identified statistically significant. For cross-sectional assessments, ASM, HGS, and walking speed tertiles were used to compare cognitive performance in male and female subjects. Categorical, normally distributed and non-normally distributed continuous variables were reported as frequencies (percentages), median (25 and 75% interquartile), and means ± SD, accordingly. Multivariate linear regression assessments probed independent associations between cognitive performance and ASM, HGS, together with walking speed. The R ‘TwoSampleMR’ package was used to conduct all MR assessments.

## Results

### Relationships between walking speed and cognitive performance

Association between walking speed and cognitive performance was evaluated based on data from 1200 total participants (50.7% male, average age: 70.61 ± 7.64 years) (Table 2). DSST scores were higher in both male and female subjects with higher walking speed tertiles (both *p* < 0.001). When conducting linear regression assessments to further interrogate this relationship (Table 3), a strong positive association between walking speed and cognitive performance in males was noted under both the unadjusted model [B = 10.27; 95% confidence interval (CI), 8.43–12.10; *p* < 0.001] together with adjusted model (B = 4.93; 95% CI, 3.06–6.80; *p* < 0.001). Walking speed was also associated with cognitive performance within females under unadjusted model (B = 10.70; 95% CI, 8.96–12.45; *p* < 0.001) together with fully adjusted model (B = 4.91; 95% CI, 3.07–6.75; *p* < 0.001).

**Table 2:** Clinical characteristics of the study participants grouped by walking speed in males and females.

Variables	Total	Male			*p* value	Female			*p* value
Tertiles		T1	T2	T3		T1	T2	T3	
Walking speed (ft/s)	0.33–5.81	< 2.78	2.78–3.40	> 3.40		< 2.59	2.59–3.22	> 3.22	
*n*	1200	204	201	203		197	195	200	
Age (years)	70.61 ± 7.64	74.25 ± 7.69	69.84 ± 7.07	67.77 ± 6.44	< 0.001	73.79 ± 8.14	70.46 ± 7.40	67.61 ± 6.28	< 0.001
BMI (kg/m^2^)	28.33 ± 5.39	27.72 ± 5.16	28.39 ± 4.59	27.62 ± 4.27	0.200	29.44 ± 6.57	29.29 ± 5.92	27.59 ± 5.31	0.003
Ethnicity, *n* (%)					0.002				0.062
Non-Hispanic White	273 (22.8)	54 (26.5)	59 (29.4)	28 (13.8)		49 (24.9)	46 (23.6)	37 (18.5)	
Non-Hispanic Black	22 (1.8)	5 (2.5)	3 (1.5)	4 (2.0)		4 (2.0)	5 (2.6)	1 (0.5)	
Mexican American	682 (56.8)	105 (51.5)	107 (53.2)	139 (68.5)		99 (50.3)	102 (52.3)	130 (65.0)	
Other Race	223 (18.6)	40 (19.6)	32 (15.9)	32 (15.8)		45 (22.8)	42 (21.5)	32 (16.0)	
PIR	2.46 ± 1.50	2.05 ± 1.25	2.52 ± 1.49	3.27 ± 1.58	< 0.001	1.77 ± 1.19	2.28 ± 1.41	2.86 ± 1.56	< 0.001
Smoking status, *n* (%)	653 (54.4)	137 (67.1)	142 (70.6)	139 (68.5)	0.743	76 (38.6)	80 (41.0)	79 (39.5)	0.898
Drinking status, *n* (%)	729 (60.8)	146 (71.6)	152 (75.6)	161 (79.3)	0.220	74 (37.6)	85 (43.6)	111 (55.5)	0.001
DSST	40.00 (27.00–53.00)	30.00 (20.00–39.00)	39.00 (26.00–50.00)	49.00 (37.00–59.00)	< 0.001	30.00 (20.00–43.00)	42.00 (33.00–53.00)	53.00 (38.00–65.75)	< 0.001

Normally distributed continuous values in the table are given as mean ± SD, non-normally distributed continuous variables are given as median (25 and 75% interquartile) and categorical variables are given as frequency (percentage).

*BMI* body mass index, *PIR* poverty income ratio, *DSST* Digit Symbol Substitution Test.

**Table 3:** Relationships between walking speed and cognitive performance analyzed by multivariable liner regression analysis.

Model	Male		Female	
	B (95% CI)	*p* value	B (95% CI)	*p* value
DSST				
Unadjusted model	10.27(8.43–12.10)	< 0.001	10.70(8.96–12.45)	< 0.001
Adjusted model	4.93(3.06–6.70)	< 0.001	4.91(3.07–6.75)	< 0.001

Adjusted Model: adjusted for age, ethnicity, household income, smoking status, drinking status.

*CI* confidence interval, *DSST* Digit Symbol Substitution Test.

### Relationships between ASM and cognitive performance

The association between ASM and cognitive performance was next assessed using data from 2885 total participants (Table 4). In line with the approach employed above, males and females were stratified into ASM tertiles. This approach revealed that individuals in the highest ASM tertile exhibited the highest average CERAD-DR, CERAD-total, AFT and DDST scores irrespective of gender (all *p* < 0.001). Linear regression assessments also investigated associations between ASM and cognitive performance (Table 5), revealing that, in males, ASM was independently associated with CERAD-DR (B = 0.06; 95% CI, 0.03–0.09; *p* < 0.001), CERAD-total (B = 0.20; 95% CI, 0.12–0.27; *p* < 0.001), AFT (B = 0.22; 95% CI, 0.15–0.29; *p* < 0.001), and DDST (B = 0.62; 95% CI, 0.41–0.82; *p* < 0.001) scores when using unadjusted models. Similarly, under adjusted models, ASM was independently associated with male CERAD-total (B = 0.17; 95% CI, 0.01–0.33; *p* < 0.001) together with DDST (B = 0.77; 95% CI, 0.42–1.12; *p* < 0.001) scores, although the same was not true for CERAD-DR (B = 0.05; 95% CI, -0.01–0.11; *p* = 0.098) or AFT (B = 0.18; 95% CI, -0.01–0.37; *p* = 0.062) scores. Under unadjusted models, the ASM of female subjects was also independently associated with CERAD-DR (B = 0.07; 95% CI, 0.04–0.10; *p* < 0.001), CERAD-total (B = 0.23; 95% CI, 0.15–0.32; *p* < 0.001), AFT (B = 0.16; 95% CI, 0.09–0.23; *p* < 0.001), and DDST (B = 0.56; 95% CI, 0.34–0.79; *p* < 0.001) scores. Following model adjustment, ASM in females remained independently associated with CERAD-DR (B = 0.07; 95% CI, 0.01–0.14; *p* < 0.05), AFT (B = 0.25; 95% CI, 0.10–0.40; *p* < 0.01) and DDST (B = 0.94; 95% CI, 0.51–1.36; *p* < 0.001) scores, although the association with CERAD-total scores was no longer significant (B = 0.16; 95% CI, − 0.01 to 0.37; *p* = 0.062).

**Table 4:** Clinical characteristics of the study participants grouped by ASM in males and females.

Variables	Total	Male			*p* value	Female			*p* value
Tertiles		T1	T2	T3		T1	T2	T3	
ASM (kg)	8.43–46.76	< 23.49	23.49–26.70	> 26.70		< 15.90	15.90–18.99	> 18.99	
*n*	2885	467	467	469		494	493	495	
Age (years)	69.43 ± 6.77	70.56 ± 7.03	69.26 ± 6.63	67.87 ± 6.18	< 0.001	7.56 ± 7.08	69.44 ± 6.63	67.44 ± 5.90	< 0.001
BMI (kg/m^2^)	29.06 ± 6.36	24.84 ± 3.06	28.31 ± 2.98	33.61 ± 5.44	< 0.001	23.88 ± 3.50	28.54 ± 3.69	36.28 ± 6.55	< 0.001
Ethnicity, *n* (%)					< 0.001				< 0.001
Non-Hispanic White	1387 (48.1)	177 (37.9)	220 (47.1)	257 (54.8)		243 (49.2)	256 (51.9)	234 (47.3)	
Non-Hispanic Black	698 (24.2)	92 (19.7)	126 (27.0)	143 (30.5)		58 (11.7)	100 (20.3)	179 (36.2)	
Mexican American	260 (9.0)	48 (10.3)	57 (12.2)	33 (7.0)		41 (8.3)	48 (9.7)	33 (6.7)	
Other Race	540 (18.7)	150 (32.1)	64 (13.7)	36 (7.7)		152 (30.8)	89 (18.1)	49 (9.9)	
PIR	2.60 ± 1.60	2.52 ± 1.58	2.73 ± 1.59	2.95 ± 1.61	< 0.001	2.48 ± 1.57	2.50 ± 1.59	2.50 ± 1.61	0.979
Smoking status, *n* (%)	1462 (50.7)	299 (64.0)	298 (63.8)	300 (64.0)	0.996	154 (31.2)	205 (41.6)	206 (41.6)	0.001
Drinking status, *n* (%)	1971 (68.3)	385 (82.4)	388 (83.1)	399 (85.1)	0.521	244 (49.4)	272 (55.2)	283 (57.2)	0.040
CERAD-DR	6.00 (4.00–8.00)	5.00 (4.00–7.00)	6.00 (4.00–7.00)	6.00 (5.00–8.00)	< 0.001	6.00 (4.00–8.00)	6.00 (5.00–8.00)	7.00 (5.00–8.00)	< 0.001
CERAD-total	25.00 (21.00–30.00)	23.00 (18.00–27.00)	24.00 (20.00–28.00)	25.00 (21.00–29.00)	< 0.001	26.00 (20.00–30.00)	27.00 (22.00–31.00)	28.00 (23.00–32.00)	< 0.001
AFT	16.00 (13.00–20.00)	15.00 (12.00–19.00)	16.00 (13.00–20.00)	18.00 (14.00–21.00)	< 0.001	15.00 (12.00–19.00)	16.00 (13.00–20.00)	17.00 (13.00–20.00)	< 0.001
DSST	46.00 (33.00–58.00)	40.00 (29.00–50.00)	43.00 (33.00–55.00)	47.00 (36.00–57.00)	< 0.001	45.00 (31.75–58.00)	49.00 (36.00–62.00)	51.00 (39.00–63.00)	< 0.001

Normally distributed continuous values in the table are given as mean ± SD, non-normally distributed continuous variables are given as median (25 and 75% interquartile) and categorical variables are given as frequency (percentage).

*ASM* appendicular skeletal muscle mass, *BMI* body mass index, *PIR* poverty income ratio, *CERAD-DR* Consortium to Establish a Registry for Alzheimer’s Disease Delayed Recall Test, *CERAD-IR* CERAD Immediate Recall Test, CERAD-total score was calculated as the CERAD-IR score plus the CERAD-DR score, *AFT* Animal Fluency Test, *DSST* Digit Symbol Substitution Test.

**Table 5:** Relationships between ASM and cognitive performance analyzed by multivariable liner regression analysis.

Model	Male		Female	
	B (95% CI)	*p* value	B (95% CI)	*p* value
CERAD-DR				
Unadjusted model	0.06 (0.03–0.09)	< 0.001	0.07 (0.04–0.10)	< 0.001
Adjusted model	0.05 (-0.01–0.11)	0.098	0.07 (0.01–0.14)	0.031
CERAD-total				
Unadjusted model	0.20 (0.12–0.27)	< 0.001	0.23 (0.15–0.32)	< 0.001
Adjusted model	0.17 (0.10–0.33)	0.039	0.18 (-0.01–0.37)	0.062
AFT				
Unadjusted model	0.22 (0.15–0.29)	< 0.001	0.16 (0.09–0.23)	< 0.001
Adjusted model	0.18 (-0.01–0.37)	0.062	0.25 (0.10–0.40)	0.001
DSST				
Unadjusted model	0.62 (0.41–0.82)	< 0.001	0.56 (0.34–0.79)	< 0.001
Adjusted model	0.77 (0.42–1.12)	< 0.001	0.94 (0.51–1.36)	< 0.001

Adjusted Model: adjusted for age, ethnicity, household income, smoking status, drinking status.

*ASM* appendicular skeletal muscle mass, *CI* confidence interval, *CERAD-DR* Consortium to Establish a Registry for Alzheimer’s Disease Delayed Recall Test, *AFT* Animal Fluency Test, *DSST* Digit Symbol Substitution Test.

### Relationships across HGS and cognitive performance

To further extend the above assessments, data from 2621 subjects probed the associations between HGS and cognitive function (Table 6). After separating males and females into three HGS tertiles, improved cognitive performance was observed with increasing HGS tertiles irrespective of gender (all *p* < 0.001). In linear regression assessments (Table 7), HGS in males was found to be independently associated with CERAD-DR (B = 0.03; 95% CI, 0.02–0.03; *p* < 0.001), CERAD-total (B = 0.08; 95% CI, 0.06–0.10; *p* < 0.001), AFT (B = 0.07; 95% CI, 0.05–0.09; *p* < 0.001) and DDST (B = 0.26; 95% CI, 0.21–0.31; *p* < 0.001) scores under unadjusted models. While these independent relationships remained significant under adjusted models for CERAD-total (B = 0.03; 95% CI, 0.00–0.05; *p* < 0.05), AFT (B = 0.03; 95% CI, − 0.01 to 0.05; *p* < 0.01), and DDST (B = 0.12; 95% CI, 0.07–0.17; *p* < 0.001) scores, the same was not true for CERAD-DR scores (B = 0.01; 95% CI, − 0.00 to 0.02; *p* = 0.05). Under unadjusted models, HGS in female subjects was also found to be independently associated with CERAD-DR (B = 0.05; 95% CI, 0.04–0.06; *p* < 0.001), CERAD-total (B = 0.15; 95% CI, 0.12–0.19; *p* < 0.001), AFT (B = 0.09; 95% CI, 0.07–0.12; *p* < 0.001) and DDST (B = 0.48; 95% CI, 0.40–0.56; *p* < 0.001), and these adjusting for possible clinical variables, ASM was still independently associated with CERAD-DR (B = 0.02; 95% CI, 0.01–0.03; *p* < 0.01), CERAD-total (B = 0.06; 95% CI, 0.02–0.10; *p* < 0.01), AFT (B = 0.06; 95% CI, 0.03–0.09; *p* < 0.001) and DDST (B = 0.24; 95% CI, 0.15–0.33; *p* < 0.001) scores.

**Table 6:** Clinical characteristics of the study participants grouped by HGS in males and females.

Variables	Total	Male			*p* value	Female			*p* value
Tertiles		T1	T2	T3		T1	T2	T3	
HGS (kg)	15.70–128.90	< 67.99	67.99–82.00	> 82.00		< 43.72	43.72–52.30	> 52.30	
*n*	2621	429	424	436		444	441	447	
Age (years)	69.41 ± 6.77	72.41 ± 7.07	69.55 ± 6.52	66.39 ± 5.36	< 0.001	73.01 ± 6.76	69.07 ± 6.18	66.10 ± 5.30	< 0.001
BMI (kg/m^2^)	29.13 ± 6.37	27.45 ± 5.55	28.23 ± 5.07	29.89 ± 5.66	< 0.001	28.55 ± 6.85	29.55 ± 7.31	30.97 ± 6.75	< 0.001
Ethnicity, *n* (%)					< 0.001				< 0.001
Non-Hispanic White	1288 (49.1)	213 (49.7)	197 (46.5)	198 (45.4)		263 (59.2)	231 (52.4)	186 (41.6)	
Non-Hispanic Black	622 (23.7)	62 (14.5)	106 (25.0)	152 (34.9)		60 (13.5)	70 (15.9)	172 (38.5)	
Mexican American	217 (8.3)	39 (9.1)	41 (9.7)	32 (7.3)		35 (7.9)	42 (9.5)	28 (6.3)	
Other Race	494 (18.8)	115 (26.8)	80 (18.9)	54 (12.4)		86 (19.4)	98 (22.2)	61 (13.6)	
PIR	2.64 ± 1.60	2.43 ± 1.54	2.71 ± 1.59	3.11 ± 1.60	< 0.001	2.20 ± 1.47	2.63 ± 1.63	2.79 ± 1.64	< 0.001
Smoking status, *n* (%)	1351 (51.5)	272 (63.4)	267 (63.0)	285 (65.4)	0.755	168 (37.8)	169 (38.3)	190 (42.5)	0.299
Drinking status, *n* (%)	1819 (69.4)	344 (80.2)	359 (84.7)	374 (85.8)	0.064	214 (48.2)	244 (55.3)	284 (63.5)	< 0.001
CERAD-DR	6.00 (5.00–8.00)	5.00 (2.00–7.00)	6.00 (4.00–7.00)	6.00 (5.00–8.00)	< 0.001	6.00 (4.00–8.00)	7.00 (5.00–8.00)	7.00 (5.00–8.00)	< 0.001
CERAD-total	25.00 (21.00–30.00)	22.00 (18.00–26.00)	24.00 (19.00–28.00)	26.00 (22.00–30.00)	< 0.001	25.00 (20.00–29.00)	27.00 (23.00–31.00)	29.00 (24.00–32.00)	< 0.001
AFT	16.00 (13.00–20.00)	15.00 (12.00–18.00)	16.00 (13.00–20.00)	18.00 (14.00–22.00)	< 0.001	15.00 (12.00–19.00)	16.00 (13.00–20.00)	18.00 (14.00–21.00)	< 0.001
DSST	46.00 (34.00–59.00)	39.00 (28.00–49.00)	44.00 (32.00–55.00)	48.00 (37.00–59.00)	< 0.001	42.50 (31.00–54.00)	50.00 (38.00–63.00)	56.00 (44.00–66.00)	< 0.001

Normally distributed continuous values in the table are given as mean ± SD, non-normally distributed continuous variables are given as median (25 and 75% interquartile) and categorical variables are given as frequency (percentage).

HGS hand grip strength; BMI, body mass index; PIR, poverty income ratio; CERAD-DR Consortium to Establish a Registry for Alzheimer’s Disease Delayed Recall Test; CERAD-IR CERAD Immediate Recall Test; CERAD-total score was calculated as the CERAD-IR score plus the CERAD-DR score. AFT Animal Fluency Test; DSST Digit Symbol Substitution Test.

**Table 7:** Relationships between HGS and cognitive performance analyzed by multivariable liner regression analysis.

Model	Male		Female	
	B (95% CI)	*p* value	B (95% CI)	*p* value
CERAD-DR				
Unadjusted model	0.03 (0.02–0.03)	< 0.001	0.05 (0.04–0.06)	< 0.001
Adjusted model	0.01 (-0.00–0.02)	0.050	0.02 (0.01–0.03)	0.006
CERAD-total				
Unadjusted model	0.08 (0.06–0.10)	< 0.001	0.15 (0.12–0.19)	< 0.001
Adjusted model	0.03 (0.00–0.05)	0.021	0.06 (0.02–0.10)	0.002
AFT				
Unadjusted model	0.07 (0.05–0.09)	< 0.001	0.09 (0.07–0.12)	< 0.001
Adjusted model	0.03 (0.00–0.05)	0.021	0.06 (0.03–0.09)	< 0.001
DSST				
Unadjusted model	0.26 (0.21–0.31)	< 0.001	0.48 (0.39–0.56)	< 0.001
Adjusted model	0.12 (0.69–0.17)	< 0.001	0.24 (0.15–0.33)	< 0.001

Adjusted Model: adjusted for age, ethnicity, household income, smoking status, drinking status.

*HGS* hand grip strength,* CI* confidence interval,* CERAD-DR* Consortium to Establish a Registry for Alzheimer’s Disease Delayed Recall Test,* AFT* Animal Fluency Test,* DSST* Digit Symbol Substitution Test.

### The impact of genetically predicted sarcopenia-related traits on cognitive performance

Next, MR assessments were conducted based on IVW meta-assessments performed under a random-effects model. For these IVW assessments (Table 8), genetically predicted walking speed and ALM were both positively associated with cognitive performance, with Beta coefficients of 0.34 [standard error (SE) = 0.09; *p* < 0.001] and 0.07 (SE = 0.01; *p* < 0.010), accordingly. Similarly, lower HGS was negatively correlated with cognitive performance, with a Beta coefficient of -0.064 (SE = 0.03; *p* < 0.05). Scatter plots produced to examine relationships between sarcopenia-associated traits and cognitive performance also yielded similar results (Supplementary Figure S1).

**Table 8:** Associations between sarcopenia-related traits and cognitive performance using Mendelian randomization.

Exposures	Outcomes	IVW		MR Egger		Weighted median	
		Beta (SE)	*p*	Beta (SE)	*p*	Beta (SE)	*p*
Walking pace	Cognitive performance	0.34 (0.09)	< 0.001	0.39 (0.41)	0.349	0.25 (0.10)	0.012
Appendicular lean mass	Cognitive performance	0.07 (0.01)	< 0.001	0.03 (0.02)	0.277	0.06 (0.01)	< 0.001
Low hand grip strength	Cognitive performance	-0.06 (0.03)	0.017	-0.24 (0.07)	0.004	-0.08 (0.03)	0.005
Cognitive performance	Walking pace	0.07 (0.01)	< 0.001	0.01 (0.05)	0.865	0.06 (0.01)	< 0.001
Cognitive performance	Appendicular lean mass	0.06 (0.02)	< 0.001	-0.02 (0.07)	0.753	0.05 (0.02)	0.006

		OR (95% CI)	*p*	OR (95% CI)	*p*	OR (95% CI)	*p*
Cognitive performance	Low hand grip strength	0.91 (0.83–1.00)	0.054	0.84 (0.54–1.31)	0.448	0.89 (0.80–1.00)	0.042

*IVW* inverse-variance weighted, *MR* Mendelian randomization, *CI* confidence interval, *OR* odds ratio.

### The impact of genetically predicted cognitive performance on sarcopenia-related traits

IVW assessments further revealed a positive association between genetically predicted cognitive performance and both walking speed and ALM, with corresponding Beta coefficients of 0.07 (SE = 0.01; *p* < 0.001) and 0.06 (SE = 0.02; *p* < 0.001). Each 1-standardized sore higher cognitive score was causally associated with a 0.07-SD higher walking speed and a 0.06-SD higher ALM. Nil major association between genetically predicted cognitive performance and low HGS was noted, having odds ratio of 0.91 (95% CI, 0.83–1.00; *p* = 0.054) (Table 8). Scatter plots also confirmed these significant correlative relationships between cognitive performance and sarcopenia-related traits (Supplementary Figure S2).

## Discussion

The present analysis was conducted with the goal of examining the relationships between sarcopenia-related traits and cognitive performance. This approach revealed strong associations between sarcopenia-related traits and cognitive performance in both males and females. Strikingly, there appeared to be a bidirectional relationship between poor sarcopenia-related variables and poor cognitive performance.

In cross-sectional assessments, all three analyzed sarcopenia-related traits were significantly associated with cognitive performance irrespective of participant gender. For example, in a particular cross-sectional investigation enrolling 422 community-dwelling older adults, lower walking speed was found to be associated with reduced cognitive performance^27^, and gait speed at baseline has been shown to predict cognitive decline in older adults over a 1-year follow-up period [^28^]. In another report, cognitive decline over the course of follow-up was found to be related to reductions in gait speed independent of cognitive status at baseline^29^. Reductions in muscle mass are a core component of sarcopenia, and one analysis conducted in older women revealed a close relationship between cognitive function and muscle mass, whereas this same relationship was not evident for bone mineral density (BMD)^30^. In contrast, a previous study focused on older adults in China found that muscle mass was significantly linked to cognitive performance only in males and not in females^31^. HGS serves as a means of measuring muscle strength and an indicator of sarcopenic status. In two prior cross-sectional assessments, older adults with low HGS were found to exhibit poorer cognitive performance for domains including executive function, attention, and memory^32,33^. Overall, the present results highlight strong correlative relationships between each of these sarcopenia-related traits and participant cognitive performance.

These assessments revealed that sarcopenia-associated traits were not independently related to each cognitive performance test following adjustment for potential covariates. One potential explanation for this finding is that each cognitive performance test may reflect different aspects of brain function. The other potential explanation may be that despite being relatively simple acts, walking speed and HGS still necessitate coordination and the integration of several different regions of the brain^34,35^, such that walking speed and HGS declines may be attributable to functional changes in various brain regions. The associations between sarcopenia-associated traits and cognitive performance were significant in both males and females, but overall, the associations were stronger in females than in males. This aligns well with prior results from a cross-sectional study conducted in Japan, and may be a result of biological differences in body composition, physical performance, sex hormone levels, brain volume, and other factors between males and females^36^.

Here, MR assessments demonstrated that lower walking speed, ASM, and HGS were all related to a higher risk of decreased cognitive performance. In addition to its role in the context of exercise, skeletal muscle can also perform endocrine functions through the secretion of myokines. A drop in skeletal muscle mass and function thus correlates with impaired myokine production^37^. The key myogenic factor BDNF (brain-derived neurotrophic factor) is significantly increased under conditions of muscle contraction. BDNF is capable of crossing the blood–brain barrier and promoting neurogenesis, learning, and memory, thereby contributing to improved cognition^38^. Continuous physical training in adults aged 50–70 has been shown to stimulate significant increases in serum BDNF concentrations and associated improvements in cognitive performance^39^. Physical activity has also been shown to drive enhanced expression of hippocampus BDNF, a cerebral region central to learning and memory^40^. Aberrant myokine secretion may thus underlie the associations between sarcopenia and reduced cognitive performance. Sarcopenia can contribute to the impairment of mobility and activity adherence, resulting in a reduction in cerebral blood flow in a manner that may impact cognition^41^. Lower levels of physical activity can also contribute to a higher risk of cardiovascular disease and consequent cognitive decline^42^, in addition to promoting amyloid-β and tau protein accumulation within the brain^43^. Skeletal muscle is also the core metabolic tissue where insulin functions, and the loss of both skeletal muscle mass and function can significantly promote insulin resistance^44^. Thus, insulin resistance was on the causal pathways from sarcopenia-related traits to cognitive impairment. Moreover, the cerebral cortex structure can exert as a neuroimaging biomarker predicting cognitive impairment^45^, and a MR study revealed that among sarcopenia-associated traits, ALM and HGS could affect brain cortical structure^46^. A cross-sectional study also found that in patients with MCI, low walking pace was independently associated with specific brain structural changes^47^. Multiple mechanisms may thus govern the ability of lower levels of muscle mass and function to impair cognitive function.

The present study also demonstrated that impairment cognitive function was causally associated with low walking pace and low ALM. This may be associated with the fact that both sarcopenia and cognitive dysfunction are driven in part by age-related oxidative stress and chronic low-grade inflammation^48^. Cognitive dysfunction can also contribute to further reductions in activity and dietary intake, potentially resulting in increasingly pronounced reductions in muscle mass and function among older adults^49^.

One predominant strength from this investigation was incorporating only older adults in the cross-sectional portion of this analysis, with participants having been separated based on sex to gain more nuanced insight into the relationships of interest. In addition, such cross-sectional portion from this investigation provided validation within the associations between sarcopenia-related traits and cognitive performance, while the causal nature of these relationships was further confirmed through MR assessments. Even with these strengths, this study is subject to certain limitations. For one, a physical measurement-based formula, rather than DXA, was used when measuring ASM. In addition, the HGS GWAS data were binary, potentially constraining the findings of the conducted MR assessments.

## Conclusion

In conclusion, these results suggest that lower walking speed, muscle mass, and muscle strength are all related to lower cognitive performance irrespective of gender, and that there may be a mutually reinforcing relationship among these variables. There may thus be opportunities to escape from this vicious downward spiral through interventions aimed at improving muscle mass, muscle strength, or cognitive performance, thereby improving outcomes in older individuals.

### Supplementary Information

Supplementary Information.

## Data Availability

The data that support the findings of this study are available from the corresponding author upon request.
