# Supplementary material for: The bidirectional associations between sarcopenia-related traits and cognitive performance
Source: Sci Rep. 2024 Mar 31;14:7591. doi: 10.1038/s41598-024-58416-w (PMC10981681; doi:10.1038/s41598-024-58416-w)
Supplement: Supplementary file 1 — Supplementary Information. [file 41598_2024_58416_MOESM1_ESM.docx]

**Supplementary Figure S1. Scatter plots to assess the relationships between sarcopenia-associated traits and cognitive performance**

**A**

**
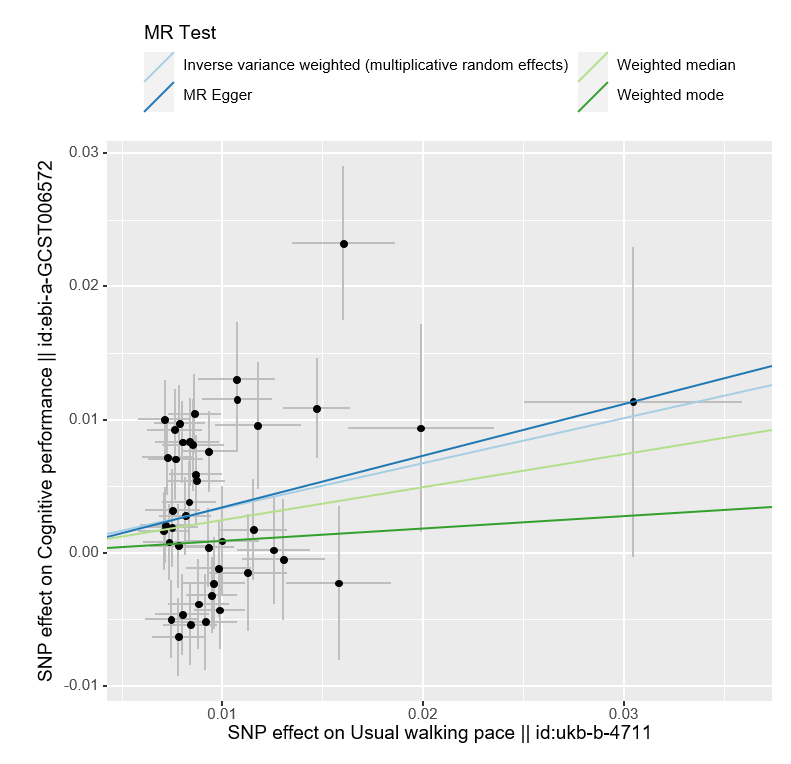
**

**B**

**
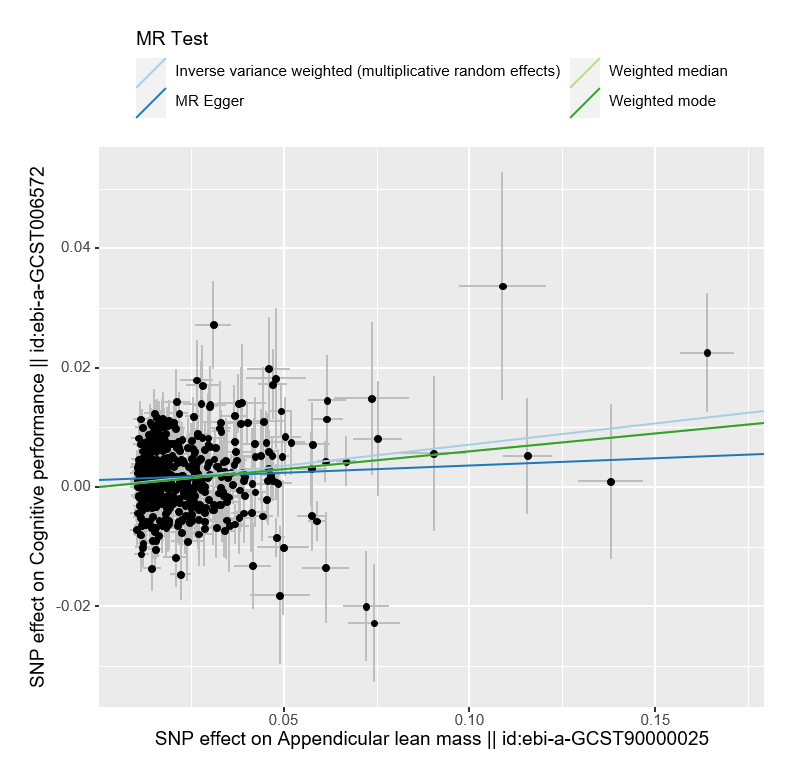
**

**C**

**
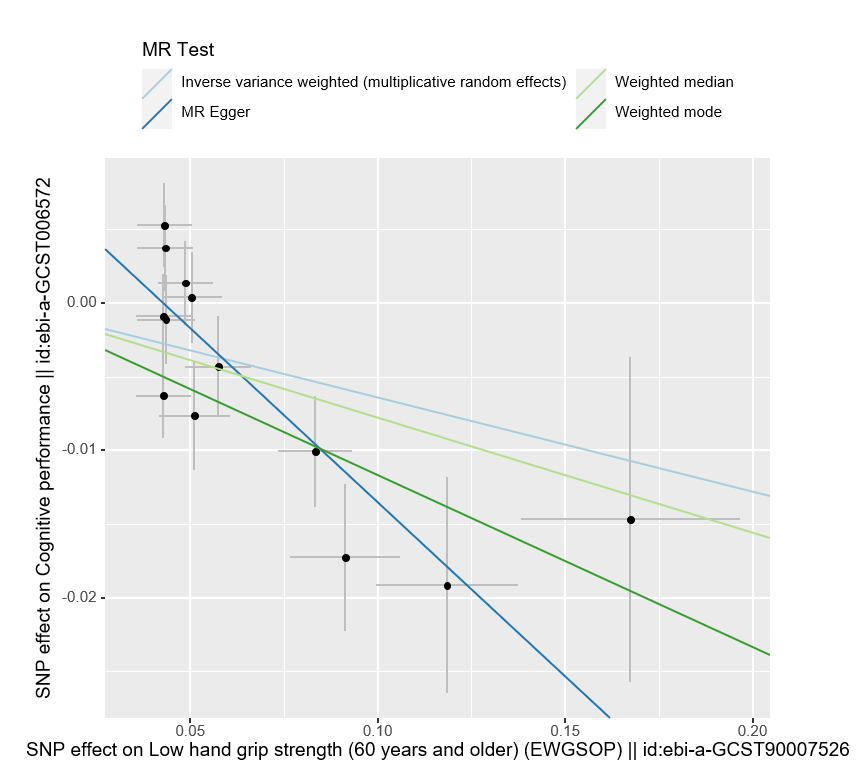
**

**Supplementary Figure S2. Scatter plots to assess the relationships between cognitive performance and sarcopenia-associated traits**

**A**

**
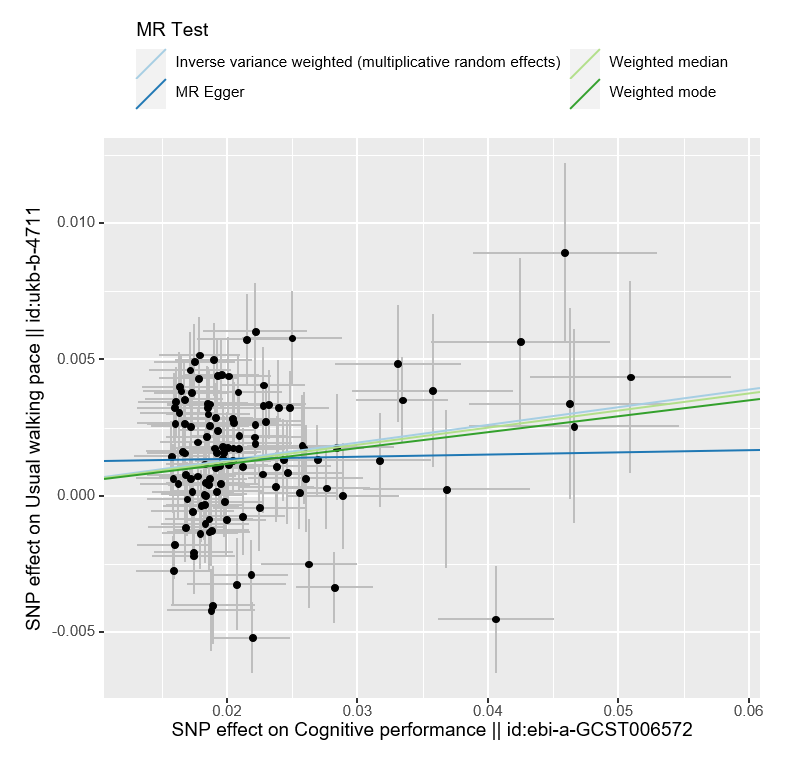
**

**B**

**
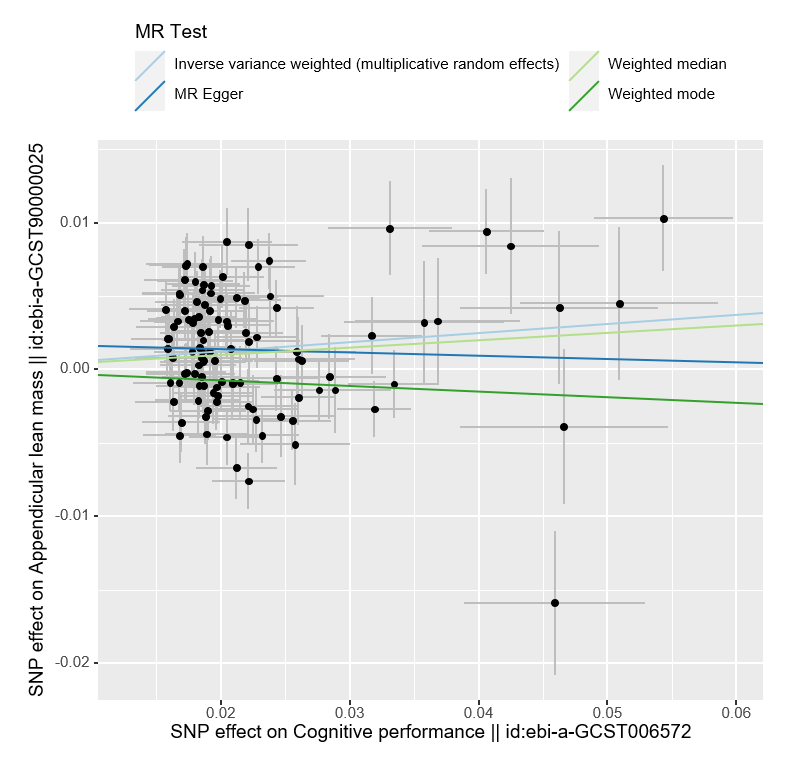
**

**C**

**
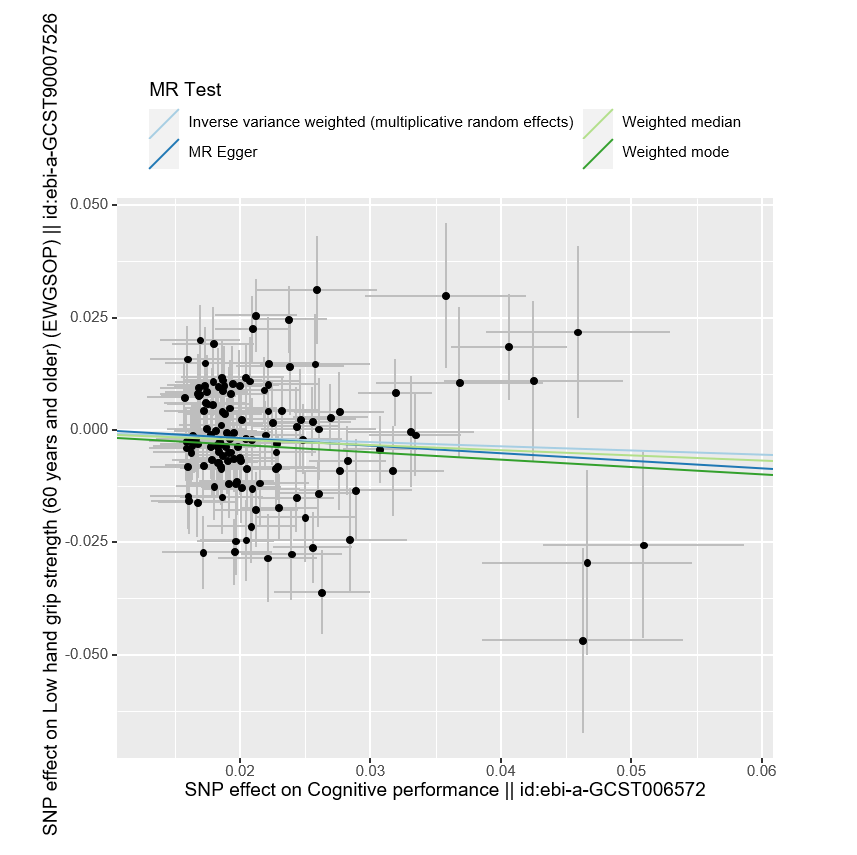
**
